# A green and verified high-performance liquid chromatographic technique for the concurrent measurement of a few veterinary drug residues in milk

**DOI:** 10.1186/s13065-025-01455-9

**Published:** 2025-04-18

**Authors:** Osama I. Abdel Sattar, Hamed H. M. Abuseada, Mohamed S. Emara, Mahmoud Rabee

**Affiliations:** 1 Pharmaceutical Analytical Chemistry Department, Faculty of Pharmacy, Al-Azhar University, Nasr City, 11751 Cairo Egypt; 2 Research and Development Department, Heliopolis University, Cairo, 11785 Egypt

**Keywords:** Milk, Imidocarb dipropionate, Flunixin meglumine, Sulfadimidine, HPLC

## Abstract

Milk is a widely consumed dietary product due to its high nutritional value. The presence of veterinary drug residues in milk constitutes a potential risk to human health and undesirable effects on consumers. In this study, a chromatographic method was developed and optimized for quantitative analysis of imidocarb dipropionate (IMD), flunixin meglumine (FNM), and sulfadimidine (SDD) residues in milk. These drugs are used together as a combination therapy for the management of anaplasmosis in cattle. The chromatographic separation was performed using an ODS Hypersil C18 column with UV detection at 270 nm. The mobile phase consisted of 0.05 M phosphate buffer, pH 3: acetonitrile: methanol (55:30:15, by volume), with a flow rate of 1 mL/min. Before analysis, a protein precipitation procedure was performed to extract the studied drugs from milk by using methanol as an extractor/deproteinization agent. The proposed method was successfully employed to quantify the studied drug residues in cattle milk samples within and after their withdrawal periods. The developed method was statistically compared with reported methods, demonstrating no significant difference in terms of accuracy and precision. Greenness and environmental impact were also evaluated for the proposed procedure, verifying it was a green and eco-friendly analytical method.

## Introduction

The use of veterinary drugs in food-producing animals can generate residues in animal-derived products (meat, milk, eggs, and honey) and pose a health hazard to the consumer. The major public health significances of drug residues are the development of antimicrobial drug resistance, hypersensitivity reaction, carcinogenicity, mutagenicity, teratogenicity, and disruption of intestinal normal flora [1]. There are many factors influencing the occurrence of veterinary drug residues in animal products, but the most probable factors are inappropriate usage and failure to keep the withdrawal period [2]. Consequently, the World Health Organization (WHO), in cooperation with the worldwide governments, set a series of legislations to regulate the use of those drugs [3, 4].

The control of veterinary drug residues in middle- and low-income countries represents high challenges. The limited research resources of their regulatory laboratories and the lack of awareness among farmers are the major factors for the misuse and non-compliance behavior with the veterinary drug usage standards [5]. Most of the published literature dealing with the veterinary drug residues analysis is based primarily on liquid/gas chromatography with MS/MS detection [6]. The MS/MS detection is costly and not commonly available in most regulatory laboratories in limited-income countries.

In this study, three of the most frequently used veterinary drugs were selected to be analyzed in milk samples. Their names were imidocarb dipropionate (IMD), flunixin meglumine (FNM), and sulfadimidine (SDD). These drugs are used together as a combination therapy for the management of anaplasmosis in cattle [7–9]. Anaplasmosis is a worldwide hemolytic disease in cattle, caused by a gram-negative obligatory intracellular bacterium called *Anaplasma marginale* [10]*.* This disease is characterized by anemia and jaundice, alongside many other gastrointestinal symptoms. This hemolytic disorder results in a significant economic loss to both dairy and beef industries [11].

**Fig. 1:**
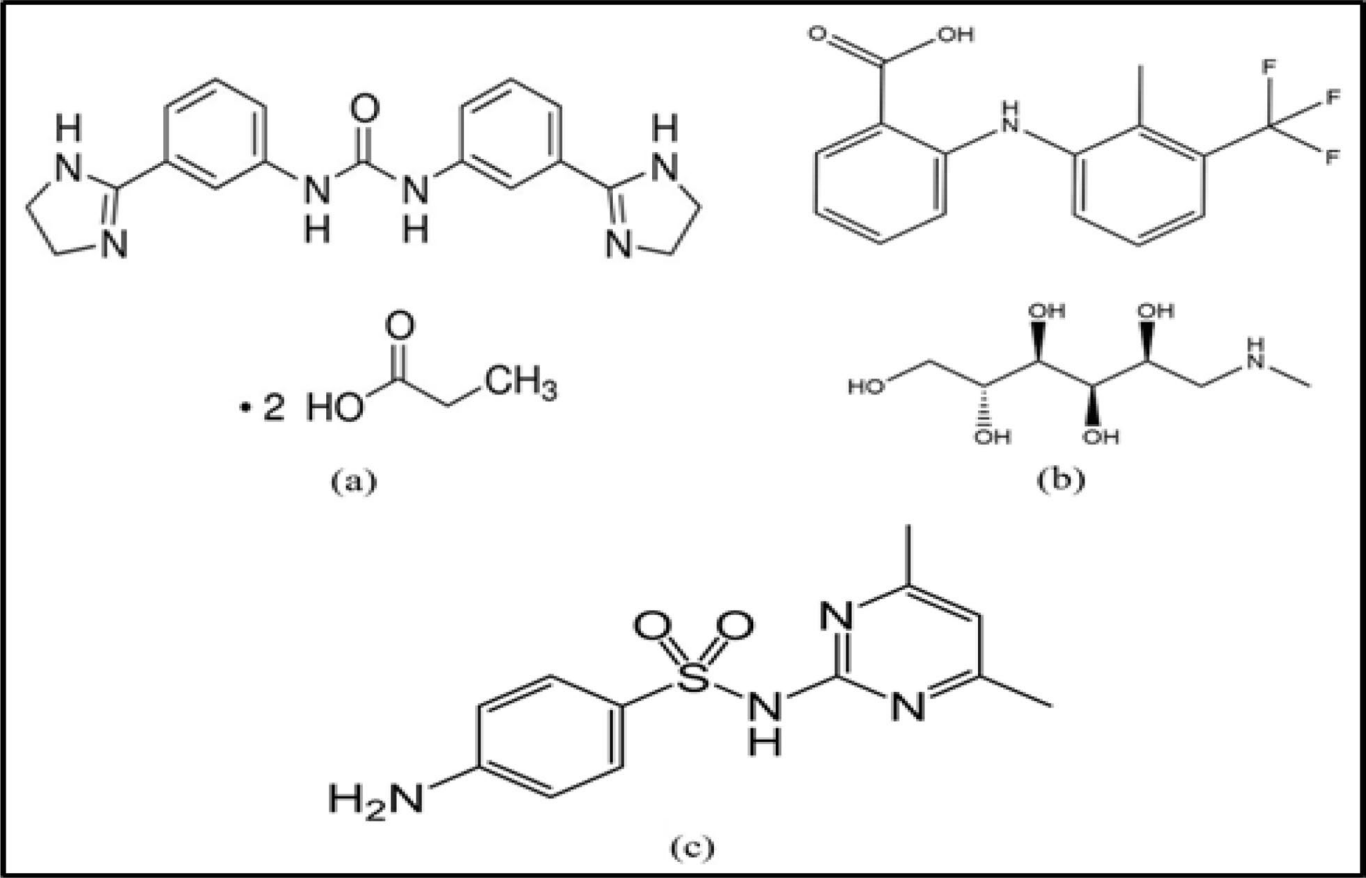
Chemical structures of (**a**) IMD, (**b**) FNM, and (**c**) SDD

IMD (Fig. 1a) is a carbanilide derivative drug, chemically known as [1,3-bis [3-(4,5-dihydro-1h-imidazol-2-yl) phenyl] urea] in the form of dipropionate salt [12]. It is a chemotherapeutic agent with antiprotozoal activity [13]. IMD is available in the form of a sterile clear aqueous injectable solution, and the dose for managing anaplasmosis is a single dose of 3 mg/kg body weight via the intramuscular (IM) route [7]. IMD is excreted unchanged in milk with a withdrawal period of 6 days [7]. Different methods were reported for determination of IMD, including HPLC [13], LC MS/MS [14], and potentiometric determination [15, 16].

FNM (Fig. 1b) is a nicotinic acid derivative drug, is chemically known as (2-[[2-methyl-3-(trifluoromethyl)-phenyl] amino]−3-pyridinecarboxylic acid compounded with 1-deoxy-1-(methylamino)-d-glucitol (meglumine salt). It is a non-steroidal anti-inflammatory drug (NSAID) used in veterinary medicine to relieve pain and inflammation in acute and chronic disorders. It blocks some part of the cyclooxygenase enzyme pathway and thereby suppresses the synthesis of several chemical mediators of inflammation [17]. FNM is used as an analgesic for anaplasmosis-positive cases in the form of IM injection at a dose of 1.1–2.2 mg/kg body weight every 12 h [8]. FNM is excreted unchanged in milk with a withdrawal period of 36 h [18]. Different techniques were reported for FNM determination, like spectrophotometry [19] and HPLC [20].

SDD (Fig. 1c) is 4-amino-*N*-(4, 6-dimethyl-2-pyrimidine) benzene sulfonamide. It is a broad-spectrum antibacterial drug that belongs to the sulfonamide class. It is widely used in veterinary practice to treat livestock diseases such as gastrointestinal and respiratory tract infections [21]. SDD is used with IMD as a supportive treatment in anaplasmosis-positive cases, in the form of IM injection, at a dose of 200 mg/kg body weight every 24 h for 5 days [9]. SDD is excreted unchanged in milk with a withdrawal period of 5 days [22]. Various methods were reported for SDD determination, such as LC–MS/MS [23] and HPLC [24].

A sample preparation step, prior to analysis, is required by most of the proposed methods to isolate the target analytes from the complex matrices like milk [25]. Classical extraction procedures, such as liquid–liquid extraction, have the disadvantages of consuming large volumes of solvents and being extremely low in selectivity. Protein precipitation extraction is considered to be an effective alternative, as it has the advantage of being simple, cost-effective and suitable for low-income countries [26].

The main purpose of this study was to develop a validated, sensitive, and eco-friendly HPLC method. This accurate method is suitable for simultaneous determination and routine quality inspection of the studied drugs in milk samples. The developed method was validated according to ICH guidelines [27]. Prior to analysis, a protein precipitation procedure using methanol as an extractor/deproteinization agent was performed to extract the target drugs from milk. The proposed method was employed to quantify the studied drug residues in cattle milk samples within and after their withdrawal periods. The developed method was statistically compared with the reported methods of each drug. The proposed method was also evaluated for its greenness and environmental impact by a well-known assessment tool called Analytical Eco-Scale.

## Experimental

### Instruments

An HPLC system model 1100 (Agilent, USA), equipped with a G1311A quaternary pump, G1329B auto-sampler, and G1314A variable wavelength (UV) detector, was used. The chromatographic separation was performed on an ODS Hypersil C18 column (250 × 4.6 mm, 5 µm particle size). A Bio-Base pH meter model PHS-3BW (China) was used for the measurement of pH. A Bio-Base centrifuge model BTBK-12HRT3 (China) was used in the protein precipitation extraction procedure.

## Materials

### Pure standards

Pharmaceutical-grade IMD (98.86%), FNM (99.37%), and SDD (100.23%) were kindly supplied by Pharma-Swede Company (Egypt). Their percentages of purity were according to the certificates of analysis from the manufacturer.

### Milk samples

Thirty fresh raw milk samples were collected from five mature lactating cattle (3 buffalo and 2 cows). Their age and weight were almost 2 years and 535 ± 25 kg, respectively. They were kept in a tie-stall housing system of a farm located in Sharqia Governorate, Egypt. The chosen cattle were anaplasmosis-positive cases and were receiving the combination therapy of the studied drugs in their recommended doses by a veterinarian. The milk samples (25 mL) were collected within and after the withdrawal period of each drug in sterile plastic screw caps and then stored at – 20 °C until laboratory analysis. A veterinarian did the sample collection. Before analysis, they were filtered through 0.45-µm nylon membrane filters to eliminate the suspended matter. During the method development and validation, organic milk was used to ensure the absence of any other drug residues that may cause potential matrix effects.

### Chemicals and reagents

Acetonitrile and methanol HPLC grade were purchased from Honeywell (Germany); water HPLC grade was obtained from LiChrosolv (Germany). Potassium dihydrogen phosphate was obtained from Sigma Aldrich (Germany). Ortho-phosphoric acid (85%) was purchased from EL-NASR pharmaceutical chemicals (Egypt). All used chemicals and reagents were of analytical grade or higher.

### Standard solutions

Stock solutions of IMD, FNM & SDD were prepared by dissolving 50 mg of each drug in 50 mL methanol to prepare a solution with a concentration of 1 mg/mL = 1000 µg/mL. Working standard solutions of each drug were freshly prepared by dilution from their stock solutions (5 mL in 50 mL) with methanol to obtain a concentration of 0.1 mg/mL = 100 µg/mL.

## Procedures

### Samples preparation

Sample preparation was performed using a protein precipitation extraction procedure. In a 50 mL centrifugation tube, 10 mL of a milk sample was homogenized using an ultrasonic homogenizer for 2 min. Then, 2 mL of methanol was added to precipitate milk proteins. The resulting mixture was then vortexed for 5 min and centrifuged at 8000 rpm for 15 min at 6 °C. It was observed that the low temperature enhances the protein precipitation process. The resulting supernatant was then filtered and transferred into clean tubes. The prepared samples were then analyzed under the described chromatographic conditions.

### Chromatographic conditions

HPLC chromatographic separation was achieved using an ODS Hypersil C18 column (250 × 4.6 mm, 5 µm particle size), using an isocratic elution of a mobile phase consisting of 0.05 M phosphate buffer, pH 3: acetonitrile: methanol (55: 30: 15, v/v/v), with a flow rate of 1 mL/min. Analysis was employed at ambient temperature, and detection was carried out at 270 nm. The injection volume was 20 µL.

### Validation of the procedures

#### Calibration curve construction (linearity)

Ten milliliters of each milk sample were mixed/spiked with 25 mg of each studied drug powder. The protein precipitation step was performed (as previously described), and the resulting supernatant was transferred into a 25 mL volumetric flask. The volume was completed to the mark to obtain a stock standard solution of 1000 µg/mL. Then a working standard solution of each drug was prepared by making suitable dilutions (10 mL in 100 mL) to obtain a concentration of 100 µg/mL. Aliquots from each working standard solution (100 µg/mL, prepared from spiked milk samples) of the studied drugs were accurately transferred into a series of 10 mL volumetric flasks. Each flask was completed with methanol to obtain a concentration range of 0.5–60 µg/mL. These solutions were injected (20 µL) in triplicate into the HPLC system. The chromatographic conditions were employed, and the chromatograms were recorded. Calibration curves were constructed between peak areas of the studied drugs at 270 nm versus their concentrations. The acquired regression equations were used to calculate the concentration of each drug throughout the whole study.

#### Accuracy and precision

Accuracy and precision of the method were determined by applying the proposed procedure for the determination of three different concentrations (3, 8, 20 µg/mL) for the three studied drugs. Each in triplicate on the same day (intra-day precision) and in three successive days (inter-day precision). The accuracy as % Recovery (%R) and the precision as relative standard deviation (%RSD) were calculated.

#### Extraction efficiency from milk matrix

Extraction efficiency or recovery of the studied drugs was evaluated by comparing the mean peak responses of each extracted sample (prepared at three levels of concentrations of each drug, 1, 10, 50 µg/mL) to mean peak responses of three un-extracted standards of equivalent concentration. The recovery of the analytes does not have to be 100.0%, but the extent of recovery of an analyte should be consistent, precise and reproducible [28].

#### Limits of detection and quantitation

The following equations were used to calculate the limit of detection (LOD) and the limit of quantitation (LOQ) in compliance with ICH guidelines: LOD = 3.3 σ/Slope, LOQ = 10 σ/Slope, where σ is the regression line's y-intercept standard deviation.

#### Specificity (matrix effect)

Five randomly selected drug-free milk samples (10 mL) were processed by the similar protein precipitation extraction procedure (by adding 2 mL methanol) and analyzed to determine the extent to which the endogenous milk components may interfere with the studied drugs. The selected drug-free milk samples were from different types of milk, which include three samples of fresh raw buffalo milk, cow milk, and goat milk, and two samples of pasteurized milk from the Egyptian market.

#### Selectivity (analysis of laboratory prepared mixtures)

To evaluate the selectivity of the proposed method, five laboratory-prepared mixture solutions containing different ratios of IMD, FNM & SDD in the concentration range of 0.5–60 µg/mL were prepared by diluting and mixing different aliquots from their working standard solutions into a series of 10 mL volumetric flasks and completing to final volume with methanol. The described chromatographic conditions were employed for the prepared mixtures, and the concentrations are then calculated from the corresponding regression equations.

#### System suitability parameters

Parameters including retention time (t_R_), resolution (R_s_), tailing factor (T), capacity factor (k), selectivity factor (α), number of theoretical plates (N), and height equivalent to theoretical plate (HETP) of the resulted peaks of each drug were calculated according to ICH guidelines to determine the suitability and effectiveness of the proposed chromatographic system prior to use.

### Application of the proposed method on milk samples

After reviewing the literature, the withdrawal periods for IMD, FNM, and SDD in milk were found to be 6 days, 36 h, and 5 days, respectively [7, 8, 22]. Due to their different withdrawal periods, two milk samples were collected from each lactating anaplasmosis-positive cattle (n = 5) separately within and after the withdrawal period of each drug. A total of 30 milk samples were collected, treated, and analyzed under the described chromatographic conditions. The found concentrations in µg/mL were calculated from the corresponding regression equation of each drug.

## Result and discussion

The main purpose of this study was to develop a validated chromatographic analytical method. This accurate method is suitable for simultaneous determination and routine quality inspection of some veterinary drug residues, namely: imidocarb Dipropionate (IMD), flunixin meglumine (FNM), and sulfadimidine (SDD) in different milk samples. We overcome the vital problem of potential interference of various impurities by the proper selection of the extraction procedure and the mobile phase composition.

### Optimization of experimental conditions

**Fig. 2:**
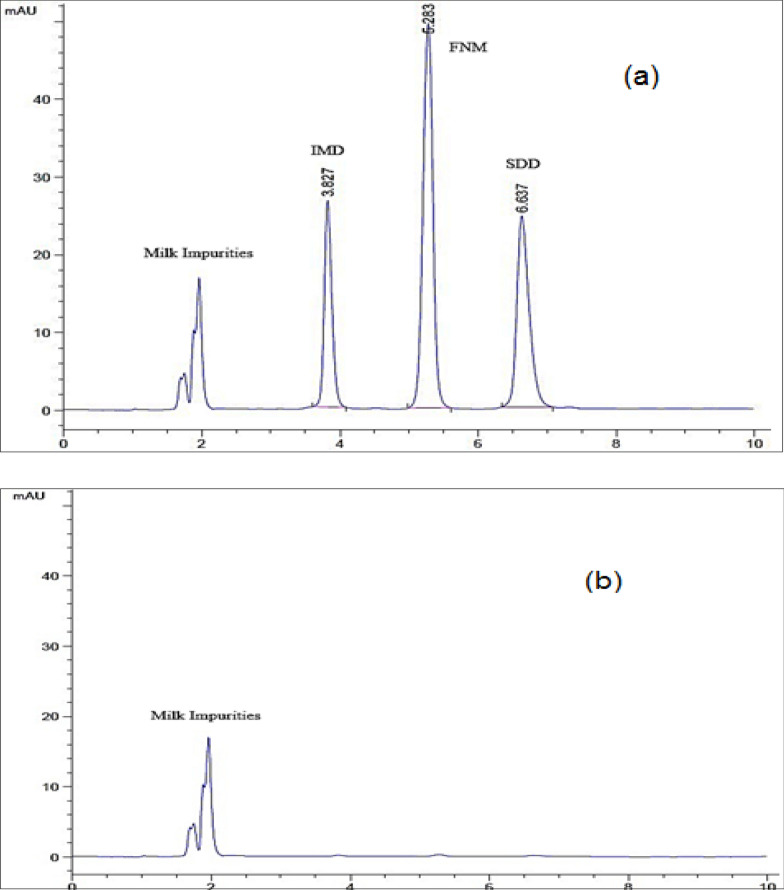
HPLC chromatograms of (**a**) retention times (t_R_) of the studied drugs, & (**b**) blank or drug free milk sample

The chromatographic conditions were optimized to get the optimum separation pattern of the studied drugs. First, two types of stationary phases were tried (Inertsil ODS C8 and ODS Hypersil C18 column), but the latter showed a more suitable resolution. For the mobile phase, first, a simple mixture of acetonitrile and water (50:50) was tried and resulted in a poor separation between the studied drugs and milk impurities. The addition of methanol in the composition of the mobile phase resulted in a satisfactory separation between the studied drugs and milk impurities with a reduction of the run time to 10 min. The use of 0.05 M phosphate buffer, pH 3, instead of water offers better resolution between the studied drugs and also better peak shape and symmetry. The absolute separation was accomplished by using 0.05 M phosphate buffer, pH 3: acetonitrile: methanol (55: 30: 15, v/v/v) as a mobile phase, which achieves excellent resolution, and sharp symmetrical peaks. The run time was 10 min, with a flow rate of 1 mL/min; increasing the flow rate (> 1 mL/min) to get a faster run time resulted in poor separation, asymmetric peaks, and an increase in column inlet pressure. The scanning wavelength was selected to be 270 nm, as it is the wavelength of maximum absorption for SDD. This selected wavelength also shows a good linearity curve for both IMD & FMN. The retention times (t_R_) were found to be 3.82 ± 0.2, 5.28 ± 0.3, and 6.6 ± 0.3 min for IMD, FNM, and SDD, respectively, as shown in Fig. 2.

## Method validation

### Linearity

**Fig. 3:**
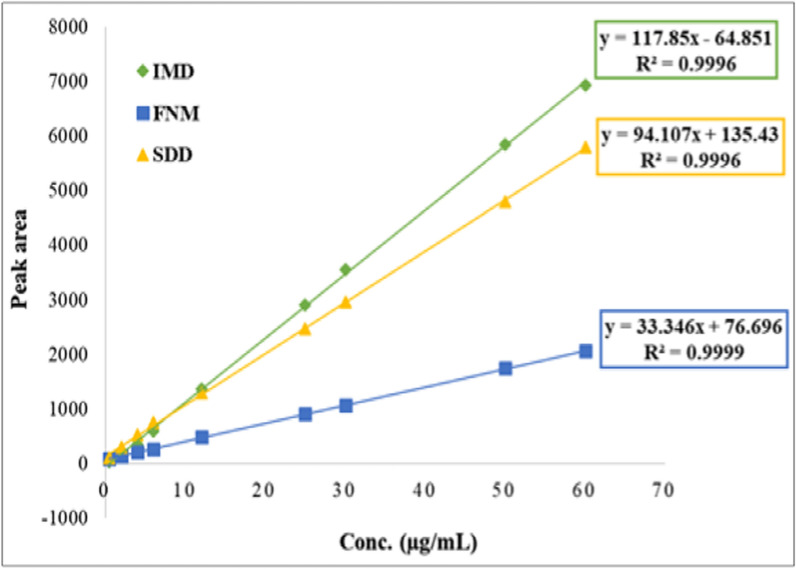
Calibration graphs of the studied drugs

Under the described experimental conditions, the calibration graphs for the three analytes were constructed by plotting peak area at 270 nm versus the corresponding concentrations of the three drugs in µg/mL. The regression plots were found to be linear over the range (0.5–60 µg/mL) for each drug, as shown in Fig. 3.

Linearity ranges, regression equations, intercepts, slopes, and squared correlation coefficients (r^2^) for the calibration data were presented in Table 1. The high value of coefficients of determination indicated good linearity of the calibration graphs.

**Table 1:** Regression and validation data of the proposed method

Parameters	IMD	FNM	SDD
λ max (nm)	270 nm		
Linearity range (µg/mL)	0.5–60	0.5–60	0.5–60
- Slope (b) - Intercept (a)	117.85	33.346	94.107
	− 64.851	+ 76.696	+ 135.43
Squared correlation coefficient (r^2^)	0.9996	0.9999	0.9996
LOD (µg/mL)	1.59 × 10^–2^	8.42 × 10^–2^	3.37 × 10^–2^
LOQ (µg/mL)	4.83 × 10^–2^	2.55 × 10^–1^	1.02 × 10^–1^

### Accuracy and precision

The accuracy of an analytical method indicates the closeness of the measurable values to the true values. The precision of an analytical method indicates the closeness of agreement between a set of measurements obtained under the prescribed conditions from multiple sampling of the same homogenous sample. The value of % Recovery confirms excellent accuracy. Moreover, the small values of % RSD indicate high precision of the method, as shown in Table 2.

**Table 2:** Accuracy and Precision of the proposed method

	Conc. (µg/mL)	Intra-day (repeatability)			Inter-day (intermediate precision)		
		Found Conc. ± SD	Accuracy^a^ (% R)	Precision^b^ (% RSD)	Found Conc. ± SD	Accuracy^a^ (% R)	Precision^c^ (% RSD)
IMD	3	2.98 ± 0.003	99.48	0.112	2.98 ± 0.005	99.37	0.152
	8	7.99 ± 0.032	99.94	0.398	7.98 ± 0.041	99.78	0.515
	20	19.92 ± 0.018	99.62	0.092	19.96 ± 0.078	99.81	0.39
FNM	3	3.00 ± 0.028	100.31	0.949	3.01 ± 0.029	100.18	0.949
	8	8.08 ± 0.009	100.35	0.107	8.07 ± 0.012	100.88	0.147
	20	20.10 ± 0.117	100.49	0.582	19.95 ± 0.157	99.75	0.787
SDD	3	3.02 ± 0.05	100.81	0.178	3.02 ± 0.005	100.89	0.173
	8	7.99 ± 0.016	99.82	0.195	8.00 ± 0.013	99.95	0.166
	20	20.06 ± 0.028	100.32	0.14	20.22 ± 0.138	101.9	0.685

^a^Average of % R of three replicates determinations

^b^Average of % RSD of three replicates determinations in the same day

^c^Average of % RSD of three replicates determinations at three successive days

### *Extraction efficiency from milk *matrix

Extraction efficiency was assessed by comparing the mean peak responses of each extracted/prepared sample (1, 10, 50 µg/mL) to the mean peak responses of three un-extracted standards of equivalent concentration. From the obtained results it can be concluded that the extraction procedure is capable of extracting 92.53% of IMD, 96.29% of FNM, and 94.91% of SDD. Extraction recoveries for all investigated drugs are given in Table 3.

**Table 3:** Mean percentage recoveries (extraction efficiency) of the target analytes from milk samples by the proposed sample preparation procedure

	Conc. (µg/mL)	Mean peak area of the extracted sample	Mean peak area of the un-extracted standard	% R	Mean % recovery (extraction efficiency)
IMD	1	52.25 ± 0.77	56.86 ± 0.77	91.89	92.53
	10	1112.06 ± 7.56	1205.72 ± 3.97	92.23	
	50	5801.91 ± 3.54	6207.39 ± 1.62	93.46	
FNM	1	112.74 ± 0.21	116.53 ± 0.43	96.74	96.29
	10	408.45 ± 1.12	425.72 ± 3.97	95.94	
	50	1738.73 ± 1.23	1805.75 ± 4.71	96.29	
SDD	1	228.94 ± 0.28	241.67 ± 1.06	94.73	94.91
	10	1080.89 ± 1.40	1137.39 ± 1.13	95.03	
	50	4814.91 ± 2.11	5070.05 ± 7.32	94.96	

### Limits of detection and quantification

Table 1 illustrates the obtained values of LOD and LOQ. The minimal values indicate good sensitivity of the proposed method.

### Specificity

Specificity of the method was evaluated by studying the HPLC chromatograms of the different types of drug-free milk samples (blank) to spiked samples. Figure 2 demonstrates that no interference between the matrix endogenous substances and the studied drugs was found.

### Selectivity

Selectivity was ascertained by analyzing five laboratory-prepared mixtures containing the studied drugs in different ratios. The separated drugs in mixtures were confirmed by comparing t_R_ values to those of standard solutions. The concentration & % recovery of each drug were calculated from the corresponding regression equation as shown in Table 4.

**Table 4:** Analysis of laboratory prepared mixtures by applying the proposed method

Mixtures ratio (I:F:S)	IMD			FNM			SDD		
	Claimed (µg/mL)	Found^a^ (µg/mL)	% R	Claimed (µg/mL)	Found^a^ (µg/mL)	% R	Claimed (µg/mL)	Found^a^ (µg/mL)	% R
3:5:2	3	2.97	98.87	5	5.00	99.99	2	1.99	99.59
3:6:3	3	2.97	99.01	6	5.95	99.21	3	3.02	100.73
5:2:7	5	4.96	99.22	2	1.99	99.31	7	6.95	99.32
8:5:4	8	8.06	100.74	5	5.08	101.62	4	3.99	99.63
9:5:7	9	8.95	99.49	5	5.00	100.01	7	6.96	99.47
Mean ± S.D^b^	99.47% ± 0.74			100.03% ± 0.96			99.75% ± 0.56		

^a^Average of three experiments

^b^Selectivity

### System suitability parameters

The parameters of the system suitability of the HPLC method were compared to the reference values. The results are listed in Table 5.

**Table 5:** System suitability of the proposed HPLC method

Parameters	IMD	FNM	SDD	Reference value
Retention time (t_R_) (min)	3.8 ± 0.3	5.3 ± 0.2	6.6 ± 0.3	–
Resolution (R_s_)	–	14.26	37.59	R_s_ > 1.5
Tailing factor (T)	1.00	1.09	1.00	T < 2 T = 1 for symmetric peak
Capacity factor (K)	2.47	3.80	5.03	1–10 acceptable
Selectivity factor (α)	–	1.53	2.03	α > 1
Number of theoretical plates (N) = column efficiency	38,714.86	32,959.09	137,096.16	≥ 2000
HETP = height equivalent theoretical plate (mm)	0.006	0.007	0.001	–

### Robustness

In the proposed method, the robustness was investigated by varying conditions with respect to flow rate, pH of the mobile phase and column temperature. The study was conducted at different flow rates (1.00 ± 0.1 mL/min), different pH of the mobile phase (3.00 ± 0.2), and different column temperature (25.00 ± 2 °C) units to study the effect of these changes on the response as represented in Table 6.

**Table 6:** Robustness results of the proposed method

Parameter	Exp. change	IMD			FNM			SDD		
		N^a^	T^b^	K^c^	N^a^	T^b^	K^c^	N^a^	T^b^	K^c^
Flow rate (mL/min)	1.00 + 0.1	38,684.93	0.98	2.45	32,969.14	1.08	3.79	137,156.18	0.98	5.09
	1.00–0.1	38,754.75	1.01	2.38	32,982.35	1.05	3.52	137,296.52	0.99	5.12
pH value (unit)	3.00 + 0.2	38,694.35	1.02	2.26	32,960.05	1.02	3.64	137,636.75	1.00	5.02
	3.00–0.2	38,754.41	0.99	2.45	32,983.52	0.99	3.96	137,536.76	0.98	5.07
Column Temp. (°C)	25.00 + 2	38,745.28	1.00	2.33	32,986.18	1.01	3.88	137,676.42	1.03	5.15
	25.00–2	38,754.78	1.02	2.54	32,979.14	0.98	3.79	137,475.93	1.00	5.18

^a^Number of theoretical plates

^b^Tailing factor

^c^Capacity factor

## Application

The proposed method was successfully applied to quantify the studied drug residues in cattle milk. The chosen cattle were three lactating anaplasmosis-positive buffalo and two lactating anaplasmosis-positive cows. They were receiving the combination therapy of the studied drugs in their recommended doses by a veterinarian. Two milk samples were collected from each lactating cattle separately within and after the withdrawal period of each drug. A total of 30 milk samples were collected, treated, and analyzed under the described chromatographic conditions. The found concentrations in µg/mL were calculated from the corresponding regression equation of each drug. The obtained results are provided in Table 7.

**Table 7:** Analysis of milk samples using the proposed method

Cattle	IMD		FNM		SDD	
	Found Conc. (µg/mL)		Found Conc. (µg/mL)		Found Conc. (µg/mL)	
	Before W.P^a^	After W.P^a^	Before W.P^a^	After W.P^a^	Before W.P^a^	After W.P^a^
Buffalo 1	50.45	1.06	20.79	–	10.23	–
Buffalo 2	60.54	0.97	30.24	1.02	23.54	0.95
Buffalo 3	42.25	–	10.24	–	26.45	0.89
Cow 1	72.45	1.59	35.45	1.78	12.87	–
Cow 2	32.45	–	29.78	–	18.45	0.64

^a^The withdrawal periods for IMD, FNM, and SDD were 6 days, 36 h, and 5 days, respectively

### Statistical analysis

Table 8 illustrates a statistical comparison between results obtained by applying the proposed method and the reported LC–MS/MS methods of the studied drugs. The calculated t and F values were less than the theoretical ones indicating that there was no significant difference between the proposed and the reported methods which reflects high accuracy and precision of the proposed method.

**Table 8:** Statistical comparison between the results obtained by the proposed method and the reported LC–MS/MS methods of the studied drugs

	IMD		FNM		SDD	
	HPLC	Reported method^a^	HPLC	Reported method^a^	HPLC	Reported method^a^
Mean	99.8	99.73	100.38	100.52	100.31	100.35
± SD	0.45	0.98	0.32	0.08	0.74	0.62
% RSD	0.175	0.245	0.346	0.112	0.223	0.275
Variance	0.2025	0.9604	0.1024	0.006	0.5476	0.3844
n	9	5	9	6	9	8
Student’s t- test	0.1511 (2.179)^b^		1.254 (2.160)^b^		0.121 (2.131)^b^	
F value	0.210 (6.04)^b^		0.625 (4.82)^b^		1.274 (3.73)^b^	

^a^IMD: [14], FNM: [29], SDD: [23]

^b^Figures between parentheses represent the corresponding tabulated value of t and F value at P = 0.05

### Assessment of the proposed method greenness

Greenness assessment of the proposed methods was accomplished using an assessment tool called Analytical Eco-Scale [30]. This approach is based on penalty points which are assigned to different factors included in the analytical method and finally subtracted from a base of 100. The score will be more than 75 for excellent green analysis, more than 50 for acceptable green analysis, and less than 50 for inadequate green analysis. The reagent type and amount, the amount of energy of various electrical devices, the analytical waste treatment, and the occupational hazard are all given penalty points. For more information about calculating Eco-scale scores, readers are directed to ref [30]. After assigning the penalty points for each analytical parameter, the proposed method got an Eco-Scale score of 79 out of 100, as shown in Table 9. The high Eco-scale score (> 75) indicated that our proposed method is an excellent green method.

**Table 9:** Analytical Eco-Scale technique penalty points for the proposed method

Hazard	HPLC
Reagents:	
Acetonitrile	6
Methanol	6
Potassium dihydrogen phosphate	0
Water	0
Instrument:	
Energy	1
Occupational hazard	0
Waste	8
Total Penalty Points (PPs) [19]	21
Analytical Eco-Scale total score	100–21 = 79

### Stability of the studied drugs in the milk matrix

The studied drugs were stable in the milk matrix at −20 °C up to 2 weeks, giving results agreeing with the initial assay results; after 4 weeks, milk endogenous peaks were observed, and some of them were at the same retention times of the studied drugs.

## Conclusion

IMD, FNM, and SDD are among the most widely used drugs in veterinary medicine to control diseases or to promote growth as food additives. The inappropriate usage of these drugs or not enough withdrawal period after treatment leads to the presence of residual drugs in livestock products causing negative effects on consumers. The suggested chromatographic method provides a simple, accurate, and reproducible method for quantitative analysis of these drug residues in the milk samples. No interferences from the milk matrix were observed. The proposed method was assessed for greenness and efficiency against reported methods and was found to be more ecologically safe and faster. It could be applied in the legislated routine analysis of these drugs at a lower analysis cost.

## Data Availability

All data generated or analysed during this study are included in this published article.
